# Global landscape of mRNA vaccine clinical trials: a systematic analysis of ClinicalTrials.gov data

**DOI:** 10.3389/fpubh.2026.1738942

**Published:** 2026-01-22

**Authors:** Sijia Liu, Tiange Zhou, Mengmeng Wang, Wanwan Xiang, Jiancai Wu

**Affiliations:** Institution for Drug Clinical Trials, Union Hospital, Tongji Medical College, Huazhong University of Science and Technology, Wuhan, China

**Keywords:** clinical trials, COVID-19, mRNA, mRNA vaccines, vaccines

## Abstract

mRNA vaccines, as a novel vaccine platform, have rapidly become a global research hotspot driven by the COVID-19 pandemic. This study employs a systematic analysis method based on clinical trial registries to conduct a descriptive statistical analysis of mRNA vaccine-related trials registered in the ClinicalTrials.gov database from March 2000 to July 2025. We compared characteristics such as the number of trials, geographical distribution, study type, funding sources, trial design, and indications, and used chi-square tests and Fisher’s exact tests for inter-group difference analysis. The results show that the number of mRNA vaccine clinical trials has experienced explosive growth after the pandemic, presenting obvious pandemic-driven characteristics and geographical differences. There are significant differences in registration characteristics and trial design among China, the United States, and Europe (*p*<0.01). Indications have rapidly expanded from infectious diseases to multiple fields such as tumors, autoimmune diseases, and metabolic diseases, indicating that mRNA technology is transforming from an infectious disease prevention tool into a platform technology with broad therapeutic potential. From the perspective of clinical trial registration, this study provides empirical evidence for understanding the global research status, regional strategy differences, and future development directions of mRNA vaccines. It offers insights for vaccine development planning, international regulatory coordination, and global clinical trial strategic planning, assisting researchers, enterprises, and policymakers in making optimal decisions.

## 1 Introduction

Vaccines are a key tool in public health, preventing billions of infections and saving millions of lives annually. Their origins can be traced back to China, India, and Africa before the 18th century (1). The modern concept of vaccines emerged from Edward Jenner in 1796, who pioneered the use of cowpox inoculation to prevent smallpox, laying the foundation for vaccination (2). The development of vaccines has progressed through three phases: the 18th to the mid-20th century focused on direct pathogen use. In the latter half of the 20th century, the advent of molecular biology enabled precise antigen extraction and recombination (3–5). Since the 21st century, genetic engineering technologies have propelled vaccine development into the gene regulation phase, spawning breakthrough technologies such as viral vectors, DNA, and mRNA vaccines, with mRNA vaccines emerging as the defining advancement of this era (6).

As early as 1990, Wolff et al. (7) first reported successful *in vivo* expression of mRNA following intramuscular injection of *in vitro*-transcribed mRNA. Subsequently, research by Conry et al. (8) demonstrated that mRNA encoding human tumor antigens could induce antigen-specific immune responses. Although progress was made in mRNA vaccine research, further development of mRNA-based vaccines and therapeutics was hindered by challenges such as instability due to RNase degradation. Over the past decade, optimization of mRNA constructs has improved stability while reducing immunogenicity, giving innovative mRNA vaccines several significant advantages. Compared to traditional vaccines, mRNA is non-infectious and cannot integrate into the host genome, allowing for rapid and efficient production in response to outbreaks and emerging viral variants (9, 10). Therefore, within less than a year after the COVID-19 outbreak, the U.S. FDA granted emergency use authorization to two mRNA vaccines targeting SARS-CoV-2: Spikevax^®^ (Moderna) and Comirnaty^®^ (Pfizer-BioNTech). Their development and administration have helped control the spread of the SARS-CoV-2 virus. Since the FDA approval of COVID-19 mRNA vaccines, mRNA vaccine technology has gradually expanded into broader applications (11).

Owing to its distinctive technological advantages and flexible research and development model, mRNA vaccines have become a prominent research focus in the global biopharmaceutical field, demonstrating considerable application potential across various domains such as infectious disease prevention and oncology therapeutics. Although prior studies have offered valuable references for academic discourse and industrial applications in this area. Current studies predominantly center on mRNA vaccines’ technical tenets, holistic development strategies, and critical advances, yet systematic analysis targeting the clinical trial stage remains largely insufficient. This includes the integration and analysis of dimensions such as the scale of clinical trial implementation, indication distribution, geographical differences, and trial design, all of which are the key elements bridging vaccine development and clinical translation. Therefore, this study aims to systematically describe the temporal trends, geographical distribution, trial design characteristics, and indications of the expansion of global mRNA vaccine clinical trials, summarize the current status and future trends of clinical trials in this field, and provide references for mRNA vaccine clinical trial researchers, pharmaceutical companies, and other relevant stakeholders. All these trials have been registered in the ClinicalTrials.gov database, which reflects global trends in clinical trial research and regulatory strategies.

## 2 Method

### 2.1 Data sources

This study is designed as a registry-based descriptive cross-sectional analysis of mRNA vaccine clinical trials. Global mRNA vaccine clinical trials were systematically retrieved from the ClinicalTrials.gov website.^1^ Established in 2000 by the National Library of Medicine (NLM), a component of the U.S. National Institutes of Health (NIH), ClinicalTrials.gov is the world’s first clinical trial registration database. It provides registration information for clinical trials conducted by public and private institutions worldwide, including study objectives, recruitment status, eligibility criteria, trial locations, and interventions, with data self-reported by sponsors through an online system.

This study was independently operated by two researchers who integrated and entered the project information. The researchers were required to retain all original data, and all modifications were recorded. When inconsistencies occurred, a 2–3 person group was formed to discuss and reach a unified judgment standard. All similar issues were corrected based on this standard.

### 2.2 Search strategy and selection criteria

To reduce selection bias, we searched the ClinicalTrials.gov website using multiple synonyms related to mRNA vaccines in the “Condition or Disease” - relevant search fields, such as “messenger mRNA,” “mRNA vaccines,” “RNA vaccines,” and “mRNA-based vaccines.” The relevant search fields do not include intervention fields such as title/brief description.

We included trials that met the following criteria: (1) Interventional studies with at least one mRNA vaccine listed in their intervention list. (2) Any trial registration status (including active, completed, terminated, withdrawn, and suspended). (3) First registration date between March 1, 2000 and June 30, 2025.

We excluded trials that met the following criteria: (1) Observational studies. (2) Interventions not involving mRNA vaccines. (3) Duplicate records.

**Figure 1:**
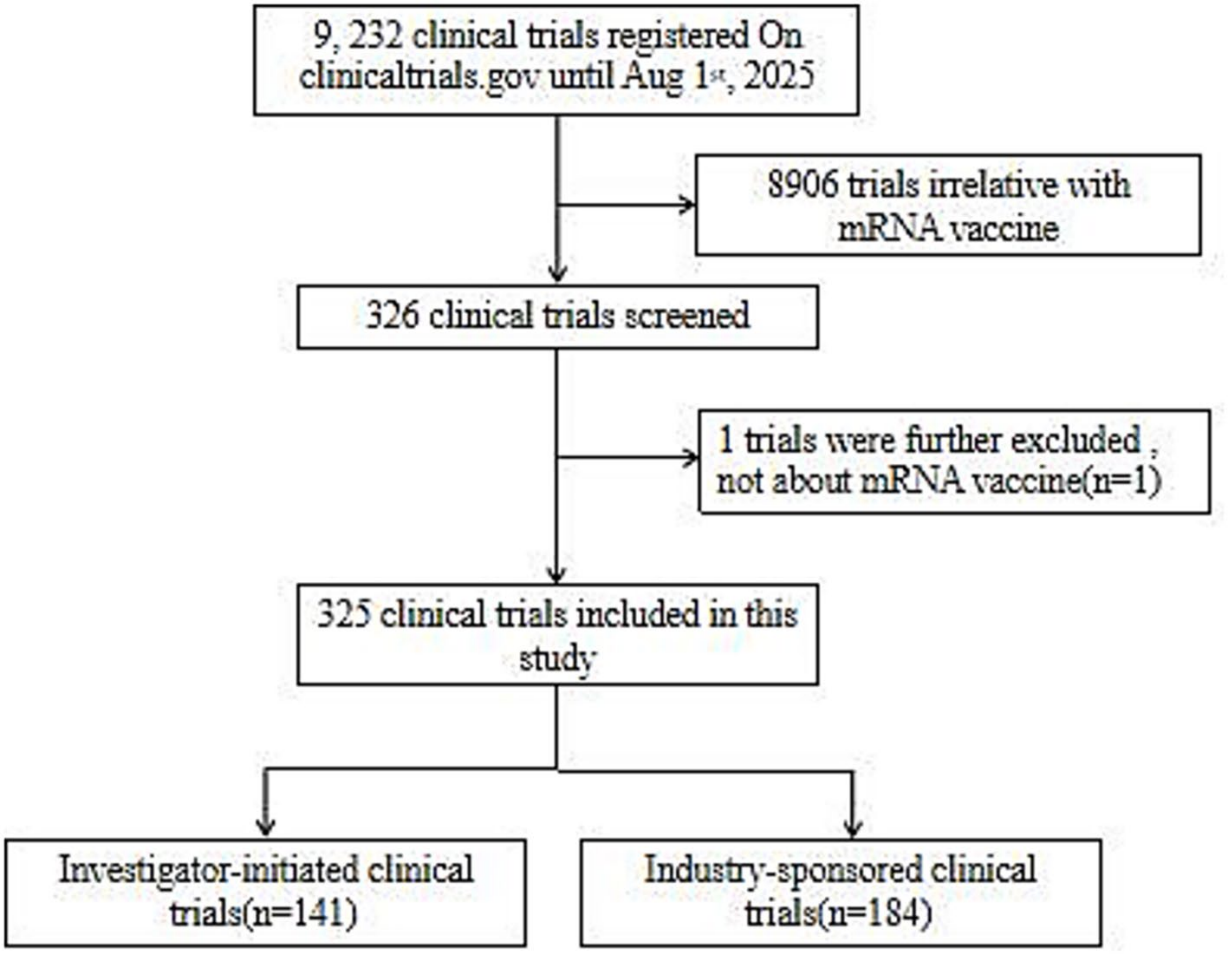
Flow diagram of the clinical trial selection process.

The following information was collected for analysis: NCT number, study title, study URL, study status, study results, conditions, interventions, sponsor, collaborators, sex, age, phases, funder type, study type, study design, start date, last update posted, and locations. Figure 1 shows the data retrieval process.

### 2.3 Statistical analysis

This study focuses on a descriptive analysis of data derived from clinical trial registries. Multivariate modeling was not the primary objective; instead, descriptive statistical methods were employed to summarize clinical trial projects using frequencies and percentages. For missing data, we first assessed its proportion and pattern: overall missing data rates were low, with variables such as “study status” and “funding type” showing the highest frequency of missing values. To mitigate systematic bias, after confirming that the missing data mechanism had a limited impact on the primary analysis results, we used listwise deletion (i.e., removing entire records containing missing values). This approach was selected based on the following rationale: (1) The study was primarily descriptive and did not involve complex parameter estimation or predictive modeling. (2) Missing data proportions were relatively low, and sufficient sample size remained after deletion to ensure statistical stability. (3) Listwise deletion avoided the additional assumptions and errors potentially introduced by imputation methods, aligning better with the conservative analytical principles of this study.

Chi-square test or Fisher’s exact test was used for intergroup comparisons, including the number of registered clinical trials, the proportion of different trial phases, indications, sponsor types, trial status, etc. All the above statistical analyses were performed using IBM SPSS Statistics Version 27.0,^2^ with a two-tailed *p*-value < 0.05 considered statistically significant (12).

### 2.4 Role of the funding source

The funding source of this study do not influence or involve in study design; collection, analysis, and interpretation of data, the writing of the report, and the decision to submit the paper for publication. All authors had full access to the data and had final responsibility for the decision to submit for publication.

## 3 Results

### 3.1 Distribution and time trends of clinical trials by sponsorship

**Figure 2:**
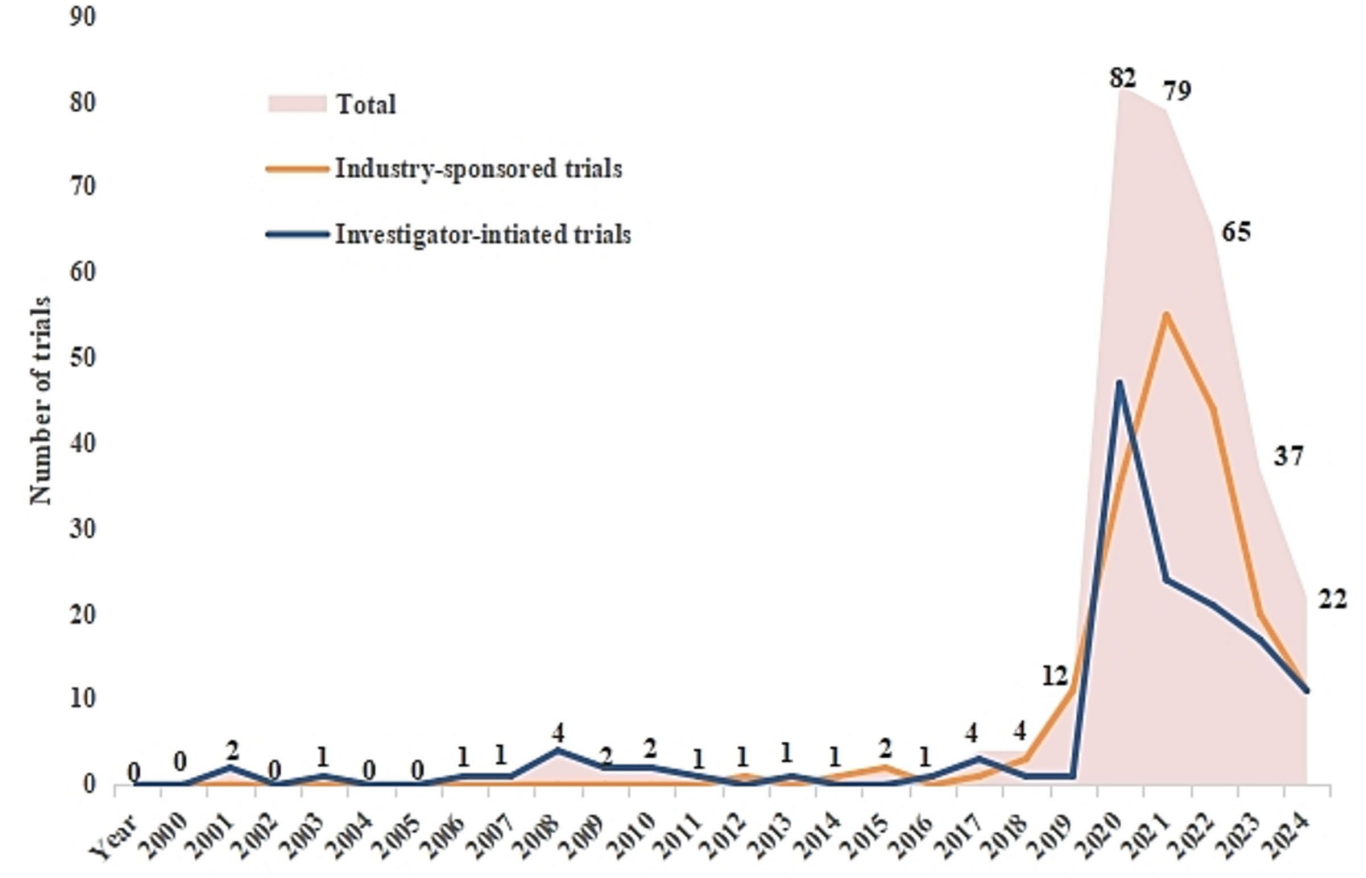
Number of annual clinical trials in mRNA vaccines by sponsor from 2000 to 2025.

This study presents a comparative analysis of the number of registered projects for industry-sponsored trials (ISTs) and investigator-initiated trials (IITs) in the mRNA vaccine field between 2000 and 2025, aiming to reveal their development trends. As shown in Figure 2, before 2020, the number of mRNA vaccine-related projects was relatively limited, with IITs predominating. In 2020, influenced by the COVID-19 pandemic, the global medical industry responded rapidly, significantly increasing investment in research and development of COVID-19-related drugs and therapies. Consequently, the number of registered mRNA vaccine projects reached a peak, with both ISTs and IITs showing significant growth. After 2020, as the development of COVID-19 vaccines gradually entered a normalized phase, the number of ISTs and IITs projects decreased noticeably. Although the overall trial scale declined from its peak, it remained consistently higher than the pre-pandemic level. The above results indicate that the COVID-19 pandemic has not only enhanced scientific community attention to and resource investment in mRNA vaccine research, but also exerted a sustained impact on the long-term scientific research progress in this field.

### 3.2 Total number of top 15 mRNA vaccine clinical trial programs worldwide

**Figure 3:**
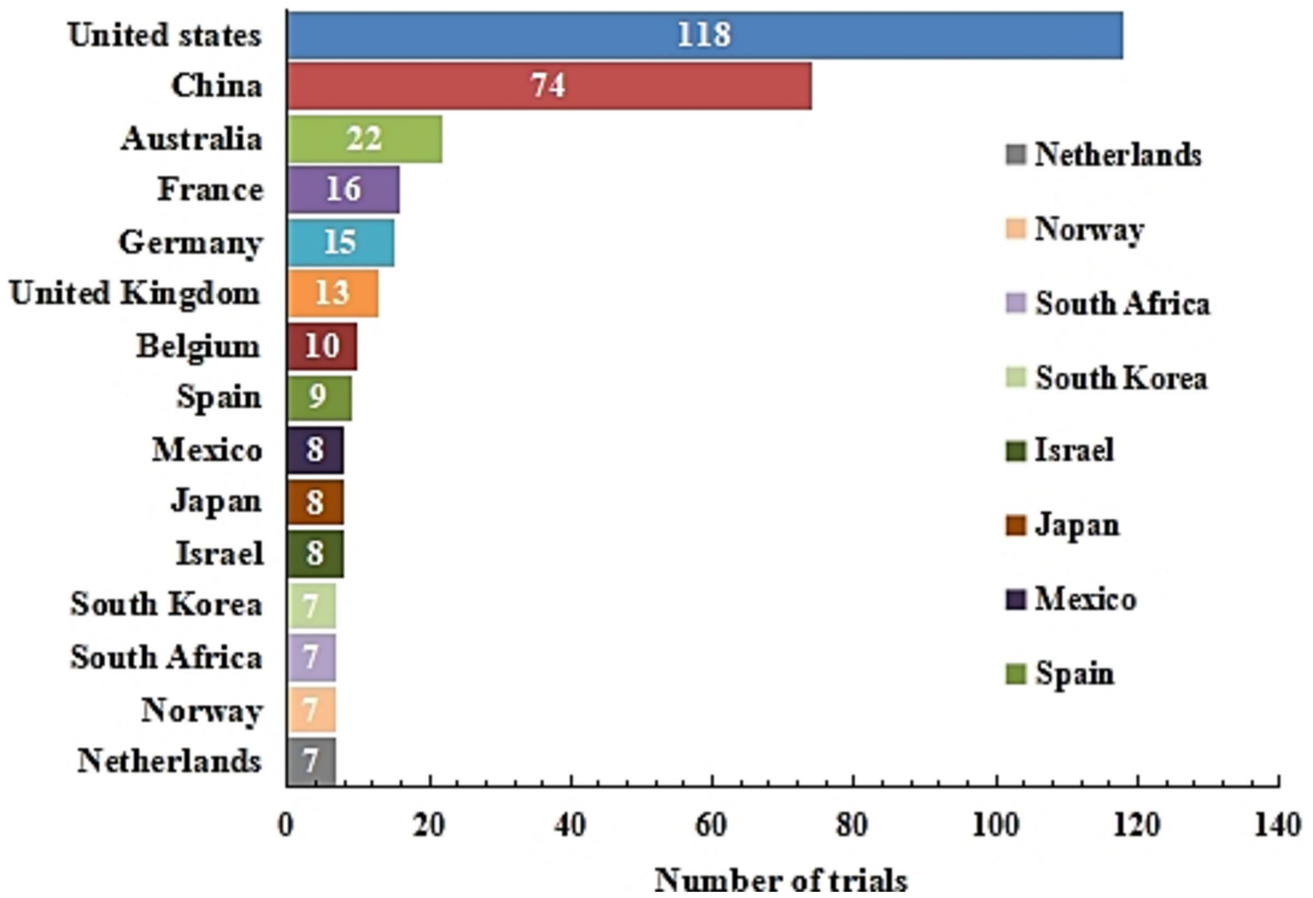
Distribution of trials in the top 15 countries around the world during 2000 to 2025.

This study presents a statistical analysis of the top 15 countries ranked by the number of registered mRNA vaccine clinical trial projects. The results show that the United States topped the list with a total of 118 trials, accounting for 35.9% of the total projects in the top 15 countries, demonstrating its dominant position in global mRNA vaccine research and development. China ranked second with 74 trials, accounting for 22.5%. Australia came in third with 22 trials, accounting for 6.7%. In terms of regional distribution, North America (including the United States and Mexico) and the Asia-Pacific region (such as China, Japan, South Korea, and Israel) showed relatively concentrated project numbers, indicating high research and development activity. Clinical research in Europe showed a more scattered pattern, with no country having a significant leading advantage. Notably, although the total number of projects in countries like South Africa is relatively limited, their long-term accumulation in infectious disease vaccine research has enabled them to maintain an important position in the mRNA vaccine research and development system (Figure 3).

### 3.3 Registration characteristics of mRNA vaccines in China, the United States, and Europe

Regarding the characteristics of trial registration, we conducted a three-group comparative analysis of mRNA vaccine clinical trials registered in China, the United States, and Europe using the chi-square test and Fisher’s exact test. The results showed that there were statistically significant differences among the three groups (China, the United States, and Europe) in terms of study type, international cooperation, study phase, and funding type (*p* < 0.01). Among them, china was dominated by domestically led interventional studies (97.3%), focusing on the early clinical phase (Phase I), with corporate funding as the main driver (58.1%). The United States also primarily conducted domestically led interventional studies (89.0%), but had a relatively high proportion of international cooperation (16.1%), and strived for balanced development across all phases from Phase I to Phase IV, with most projects funded by enterprises (74.6%). Europe had the highest proportion of international multicenter cooperative projects (77.9%), with observational studies significantly higher than those in China and the United States (23.4%). Its research phases were more diverse, with Phase III trials accounting for the highest proportion (15.6%), and the distribution of funding types was relatively extensive (Table 1).

**Table 1:** Changes of characteristics in clinical trials.

Characteristic	Total *n* (%)	Number of trials (%)			*p*-value
		China *n* (%)	United States *n* (%)	Europe *n* (%)	
Country					
Domestic	187 (69.5)	71 (96.0)	99 (83.9)	17 (22.1)	<0.001
International	82 (30.5)	3 (4.0)	19 (16.1)	60 (77.9)	
Study phase					
Early phaseI	8 (3.0)	7 (9.5)	0 (0.0)	1 (1.3)	<0.001
Phase I	71 (26.4)	28 (37.8)	30 (25.4)	13 (16.9)	
PhaseI/II	37 (13.8)	5 (6.8)	21 (17.8)	11 (14.3)	
Phase II	38 (14.1)	10 (13.5)	20 (16.9)	8 (10.4)	
Phase II/III	16 (5.9)	2 (2.7)	8 (6.8)	6 (7.8)	
Phase III	30 (11.2)	2 (2.7)	16 (13.6)	12 (15.6)	
Phase IV	16 (5.9)	2 (2.7)	9 (7.6)	5 (6.5)	
Other	53 (19.7)	18 (24.3)	14 (11.9)	21 (27.3)	
Funder type					
Industry	164 (61.0)	43 (58.1)	88 (74.6)	33 (42.9)	<0.001
GOV	5 (1.9)	0 (0.0)	0 (0.0)	5 (6.5)	
NIH	7 (2.6)	0 (0.0)	7 (5.9)	0 (0.0)	
Network	2 (0.7)	0 (0.0)	1 (0.8)	1 (1.3)	
INDIV	1 (0.4)	0 (0.0)	1 (0.8)	0 (0.0)	
Other	90 (33.5)	31 (41.9)	21 (17.8)	38 (49.4)	

### 3.4 Characteristics of mRNA vaccine trial designs in China, the United States, and Europe

In terms of trial design, we conducted a three-group comparative analysis of mRNA vaccine clinical trials registered in China, the United States, and Europe using the chi-square test and Fisher’s exact test. The results showed that there were statistically significant differences in the design characteristics of mRNA vaccine clinical trials among China, the United States, and Europe (*p* < 0.01). Regarding randomization, the United States had the highest proportion of randomized trials, accounting for 73.7%, followed by China at 52.7% and Europe at 42.9%. In contrast, China had the highest proportion of non-randomized allocation, reaching 44.6%, which was significantly higher than that of the United States (15.3%) and Europe (11.0%). From the perspective of intervention models, the parallel design was dominant in all three groups, followed by sequential design and single-arm trials. The proportion of sequential trials in the United States was significantly higher than that in China and Europe, while the proportion of single-arm trials in China was significantly higher than that in the United States and Europe. In terms of blinding methods, open-label trials accounted for the largest proportion among the three groups, followed by quadruple-blind and triple-blind trials (Table 2).

**Table 2:** Changes of trials design in clinical trials.

Study design	Total *n* (%)	Number of trials (%)			*p*-value
		China *n* (%)	United States *n* (%)	Europe *n* (%)	
Allocation					
Randomlized	159 (59.1)	39 (52.7)	87 (73.7)	33 (42.9)	0.006
Non-randomlized	76 (28.3)	33 (44.6)	18 (15.3)	25 (32.5)	
Other	34 (12.6)	2 (2.7)	13 (11.0)	19 (24.7)	
Interventional model					
Parallel assignment	151 (56.1)	40 (54.1)	70 (59.3)	41 (53.2)	<0.001
Sequential assignment	44 (16.4)	14 (18.9)	24 (20.3)	6 (7.8)	
Single group assignment	36 (13.4)	18 (24.3)	7 (5.9)	11 (14.3)	
Crossover assignment	3 (1.5)	0 (0.0)	4 (3.4)	0 (0.0)	
Other	34 (12.6)	2 (2.7)	13 (11.0)	19 (24.7)	
Masking					
Open	110 (49.0)	37 (50.0)	38 (32.2)	35 (45.5)	<0.001
Single	14 (5.2)	4 (5.4)	4 (3.4)	6 (7.8)	
Double	26 (9.7)	11 (14.9)	9 (7.6)	6 (7.8)	
Triple	34 (12.6)	8 (10.8)	22 (18.6)	4 (5.2)	
Quadruple	51 (19.0)	12 (16.2)	32 (27.1)	7 (9.1)	
Other	34 (12.6)	2 (2.7)	13 (11.0)	19 (24.7)	

### 3.5 Indications

Through a systematic review and classification of the indications of mRNA vaccine clinical trial projects, this study found that current mRNA vaccine clinical research has covered two major domains: infectious diseases and non-infectious diseases, with a clear distribution pattern of disease spectra. As shown in Table 3, within the infectious disease domain, related studies were predominantly focused on viral diseases, the number of which was significantly higher than that of bacterial diseases. Among these, respiratory viral infections emerged as a key research focus, involving coronaviruses (e.g., SARS-CoV-2), influenza viruses, respiratory syncytial viruses, and others. In addition, certain endemic viruses with potential public health threats, such as Ebola virus, Nipah virus, Zika virus, and rabies virus, have also received sustained attention. In contrast, studies on bacterial diseases were relatively fewer, mainly focusing on infectious diseases with high disease burden such as tuberculosis. In the non-infectious disease domain, mRNA vaccine research was primarily concentrated in cancer therapy, covering various solid tumors and hematological tumors including liver cancer, lung cancer, melanoma, and leukemia. Although research on other chronic diseases was more scattered, it showed a clear disease specificity, involving kidney diseases (e.g., chronic kidney disease), metabolic diseases (e.g., diabetes), autoimmune and neurological diseases (e.g., multiple sclerosis), and cardiovascular diseases (e.g., myocarditis), among others (Table 3).

**Table 3:** Classification of indicated conditions.

Classification	Diseases	Representative diseases
Infectious diseases	Virosis	Coronavirus infection, influenza, respiratory syncytial virus, HIV, cytomegalovirus (CMV), Zika virus, rabies, Papillomavirus, Nipah virus, Japanese encephalitis, Herpes Zoster, avian influenza, Ebola
	Bacteriosis	Tuberculosis, *Chlamydia trachomatis*, Lyme disease, pneumococcal infection
Non-communicable diseases (NCDs)	Various cancers	Liver cancer, lung cancer, melanoma, leukemia, prostate cancer, pancreatic cancer, solid tumors, etc.
	Kidney diseases	Chronic kidney disease, kidney transplant complications, end stage renal disease
	Metabolic disorders	Diabetes mellitus, hyperglycemia, hypoglycemia
	Autoimmune/neurological diseases	Multiple sclerosis, neuromuscular diseases
	Cardiac diseases	Myocardial injury, myocarditis, pericarditis
Other		Acne, systemic allergic reaction, fever

## 4 Discussion

Based on the registration data from the U.S. ClinicalTrials.gov database spanning from March 2000 to July 2025, this study conducted a systematic analysis of global mRNA vaccine clinical trials, aiming to reveal the registration characteristics of this field in terms of temporal growth trends, geographical distribution, trial design, and indication expansion.

From the perspective of the research and development progress of mRNA vaccines, clinical trials of mRNA vaccines exhibited a “explosive growth” feature in 2020, and the COVID-19 pandemic was undoubtedly the core driving force behind this trend. In the nearly 20 years before 2020, the number of mRNA vaccine clinical trials remained at a low level, a phenomenon closely related to the development history of mRNA technology. Although initial breakthroughs in *in vitro* transcription and expression of mRNA were achieved as early as 1990 (13–15), and its potential to induce antigen-specific immune responses was confirmed in 1995, the instability caused by RNase has long restricted technological transformation, leaving mRNA vaccines in the peripheral area of biomedical research and development without extensive industrial investment and policy support (7, 16, 17). The outbreak of the COVID-19 pandemic in 2020 completely changed this pattern. Research data show that the number of registered mRNA vaccine clinical trials reached a peak in 2020, with both Industry-Sponsored Trials (ISTs) and Investigator-Initiated Trials (IITs) achieving leapfrog growth. Behind this explosive growth lies the precise alignment between the unique advantages of mRNA technology and the needs of pandemic prevention and control. Compared with traditional vaccines, mRNA vaccines do not require pathogen cultivation; they only need to design and synthesize nucleic acid molecules based on viral gene sequences, enabling rapid research and development and large-scale production, which perfectly meets the core demand for “rapid response” of vaccines to emerging infectious diseases (18–20). The U.S. FDA urgently approved two mRNA vaccines for marketing less than a year after the pandemic outbreak (21–24). Their excellent preventive efficacy and safety not only verified the technical feasibility but also greatly stimulated the investment enthusiasm of the global scientific research and industrial communities.

In terms of the global layout of mRNA trials, the geographical distribution of global mRNA vaccine clinical trials shows significant imbalance, and the three core regions of China, the United States, and Europe have formed distinctive research and development models. The number of registered mRNA vaccine clinical trial projects in the United States ranks first in the world, accounting for 35.9% of the total number of the top 15 countries, demonstrating an absolute leading advantage, which benefits from the long-term technical accumulation and improved industrial ecosystem of the United States in the biomedical field. The United States is home to world-leading mRNA technology companies such as Moderna and Pfizer-BioNTech, and institutions like the NIH continuously provide basic research funding, forming a complete chain of “basic research - technological transformation - industrial application” (25, 26). China’s research and development characteristics are characterized by distinct “localization” and “early-stage” features. This is highly consistent with China’s strategic demand to rapidly advance local vaccine research and development and ensure public health security during the pandemic. The diversified investment subjects in China’s mRNA field also reflect the national strategic support for emerging biomedical technologies (27, 28). Europe presents a unique pattern of “high internationalization” and “diversification.” Although there is no single dominant country, the proportion of international multi-center cooperative projects in Europe is as high as 77.9%, far exceeding that of China and the United States, which is closely related to Europe’s integrated scientific research policies and tradition of cross-border cooperation (29). Such differences reflect the competition and cooperation pattern in the global biomedical field, providing data reference for policymakers to optimize the allocation of scientific research resources and improve the regulatory system.

At the same time, the significant differences in clinical trial design among China, the United States, and Europe not only reflect the different regional scientific research concepts but also are closely related to the development stage and application scenarios of mRNA technology, directly affecting the scientificity, reliability of trial results, and the efficiency of technological transformation. Essentially, the differences in trial characteristics are a comprehensive reflection of the technological development stage, research and development objectives, and regulatory environment. The importance of these findings lies in providing a “context-specific” reference for clinical trial designers. Based on the technical foundation, research and development stage, and regulatory requirements of the region, the most suitable trial design scheme can be selected to improve research and development efficiency while ensuring scientificity. Additionally, it provides a basis for regulatory authorities to formulate clinical trial guidelines. For example, for regions in the early research and development stage such as China, the design requirements for early trials can be appropriately simplified on the premise of ensuring core scientificity to accelerate technological transformation; while for mature markets such as the United States and Europe, the standardization of trial design should be continuously strengthened to ensure data reliability.

In terms of indications, mRNA vaccines have demonstrated their evolution from a “single prevention and control tool” to a “platform technology.” Before 2020, clinical trials of mRNA vaccines were mainly concentrated in the field of infectious diseases, predominantly viral diseases such as HIV, influenza, and rabies. However, due to immature technology and limited market demand, progress was slow. The COVID-19 pandemic not only promoted the outbreak of research and development on infectious disease vaccines but also activated the application potential of mRNA technology in non-infectious disease fields, rapidly expanding its indications to multiple cutting-edge areas such as tumor therapy, autoimmune diseases, and metabolic diseases (30–32).

In the field of infectious diseases, viral diseases remain the research focus, among which respiratory viral infections (such as coronaviruses, influenza, and respiratory syncytial virus) occupy a dominant position, closely related to the characteristics of these viruses such as fast transmission speed and wide harm scope (33–35). Meanwhile, endemic viruses with potential pandemic risks, such as Ebola virus, Nipah virus, and Zika virus, have also received widespread attention. Research on bacterial diseases is relatively scarce (36–38). On the one hand, the research and development of vaccines for bacterial diseases has traditionally relied on technologies such as antigen extraction and inactivation, and the adaptability of mRNA technology still needs to be explored (39). On the other hand, the pathogenesis of bacterial diseases is more complex, and the immune response induced by vaccines needs to address both the bacteria themselves and their toxins, resulting in higher technical difficulty (40). In the field of non-infectious diseases, tumor therapy has become the main research direction. The application principle of mRNA vaccines in tumor therapy is to encode tumor-specific antigens to activate the body’s own immune system to recognize and kill tumor cells (41). This “personalized immunotherapy” model has advantages such as strong targeting and low side effects. Compared with traditional tumor treatment methods (such as surgery, chemotherapy, and radiotherapy), mRNA tumor vaccines can achieve “precision strike,” with a short research and development cycle and the ability to quickly adapt to different tumor types, showing great clinical potential (42). Beyond tumors, the indications of mRNA vaccines have also extended to kidney diseases, metabolic diseases, autoimmune/neurological diseases, and cardiac diseases. Although research in these fields is relatively scattered, they all target clear clinical needs, marking that mRNA technology has broken through the traditional definition of “vaccines” and become a platform technology widely applicable to disease treatment (32, 43–46).

Despite achieving leapfrog progress in the number of clinical trials, indication expansion, and technical maturity, mRNA vaccines, as an emerging technology still in a stage of rapid development, face numerous challenges in their research and development and application. Firstly, the optimization of delivery systems and regulation of immunogenicity remain core issues. mRNA molecules themselves are unstable, easily degraded by RNase, and have certain immunogenicity, which may trigger excessive inflammatory responses. Therefore, they need to rely on delivery systems for efficient and safe delivery to target cells. The currently commonly used delivery system, Lipid Nanoparticles (LNP), still has certain limitations (47, 48). Secondly, there is a lack of long-term safety data accumulation and standardization of trial design (49). Although COVID-19 mRNA vaccines have been widely administered globally, showing good short-term safety, long-term safety still requires longer follow-up verification, such as the existence of potential immune-related adverse reactions and safety impacts on special populations (such as pregnant women, children, and immunocompromised individuals) (50). At the regulatory level, the regulatory system adapting to the characteristics of mRNA technology still needs to be improved. mRNA vaccines have a fast technological iteration speed and rapid indication expansion. For personalized tumor mRNA vaccines, the traditional model cannot meet clinical needs. Meanwhile, how to formulate scientific supplementary application and approval paths to accelerate technological transformation while ensuring safety remains a problem to be solved by regulatory authorities. In addition, differences in regulatory standards among different countries and regions may lead to obstacles in the cross-border marketing of mRNA vaccines.

In conclusion, the striking discrepancies in trial design across different regions not only reflect the divergences in the developmental stage of mRNA technology, epidemic response strategies, industrial structures, and the intensity of policy support among respective regions, but also indicate that a more inclusive and flexible collaborative framework is essential for global cooperation in mRNA vaccine research and development. On the one hand, on the premise of upholding scientific rigor, efforts should be made to explore international methodological standards tailored to distinct research and development phases, with a particular focus on balancing innovation efficiency and data quality in early exploratory trials. On the other hand, regulatory coordination mechanisms need to shift from a simplistic one-size-fits-all approach to a dynamic regulatory paradigm aligned with risk levels and clinical phases, thereby enabling countries to adopt context-specific regulatory pathways on the basis of shared risk assessments and phase-specific objectives. For vaccines targeting public health emergencies, cross-border alignment of emergency use authorization (EUA) pathways can be advanced. In contrast, for personalized applications such as tumor therapy, it is imperative to establish platforms for case data sharing and regulatory review dialogue. Only through sustained technical exchanges and institutional innovation can regional divergences be translated into complementary strengths, truly enabling mRNA technology to play a synergistic role in global public health governance. This will further accelerate the global accessibility and deployment of safe and efficacious mRNA products, ultimately maximizing the benefits of scientific research collaboration and public health outcomes.

Although this study has conducted a systematic analysis of the global landscape of mRNA vaccine clinical trials based on the mRNA vaccine clinical trial projects registered in the ClinicalTrials.gov database, it still has the following limitations: First, our analysis relies excessively on the ClinicalTrials.gov database and does not systematically incorporate other regional or national clinical trial registration databases, which thus limits the global representativeness of the research findings. Second, among the included registered trials, some lack complete trial protocols or detailed methodological descriptions, which will affect the consistency assessment during the trial implementation process. In addition, this study has not performed a systematic evaluation of the methodological quality or risk of bias of the included trials, making it impossible to conduct further assessments of the internal reliability of the trials. Finally, this study did not analyze the trial results or impacts; the conclusions are limited to the registration level and cannot be extended to the actual effects or application value of the trials.

## Footnotes

1
https://clinicaltrials.gov

2
https://www.ibm.com/products
